# Clinical characteristics, management and outcomes in patients with juvenile dermatomyositis requiring admission in pediatric intensive care unit

**DOI:** 10.1186/1546-0096-12-S1-P277

**Published:** 2014-09-17

**Authors:** Alix Besancon, Cyril Gitiaux, Karine Brochard, Nicole Brousse, Pierre Quartier, Olivier Goulet, Remi Salomon, Laurent Dupic, Christine Bodemer, Brigitte Bader-Meunier

**Affiliations:** 1 Paediatric Rheumatology, Necker -enfants Malades Hospital, Paris, France; 2 Paediatric Neurology, Necker -enfants Malades Hospital, Paris, France; 3 Paediatric Rheumatology, Hopital des enfants, Toulouse, France; 4 Anatomopathology, Necker -enfants Malades Hospital, Paris, France; 5 Paediatric gastro-enterology, Necker -enfants Malades Hospital, Paris, France; 6 Pediatric Nephrology, Necker -enfants Malades Hospital, Paris, France; 7 Paediatric Reanimation, Necker -enfants Malades Hospital, Paris, France; 8 Dermatology, Necker -enfants Malades Hospital, Paris, France

## Introduction

Juvenile dermatomyositis (JDM) are potentially life-threatening.

## Objectives

We report 11 cases of severe JDM admitted in intensive care unit (ICU) to determine their early severity signs and outcomes.

## Methods

We performed a retrospective study of cases of JDM admitted in ICU in 2 pediatric rheumatology centers (Paris, Toulouse) from 2005 to 2013, and compared them to the JDM patients who did not require ICU.

## Results

11/116 DMJ (9.3%) (8 girls and 3 boys, median age at diagnosis : 9.0± 3.1 years) were admitted in ICU for digestive involvement (2 with digestive perforation after pulse corticosteroids) (4 patients), bradycardia (1 patient), cardiac arrest (1 patient), hypoxemic pneumonia (1 patient), PRES syndrome due to cyclosporine (1 patient), thrombotic microangiopathy (TMA) (2 patients) and anaphylactic shock due to Rituximab (1 patient). The incidence of some clinical and biological manifestations differed from severe patients to patients with mild JDM: hyponatremia (9), hypoalbuminemia (9), generalized edema (6), anemia (hemoglobin value <8 g/dL) (8), abdominal pain (7), thrombocytopenia (platelet count : 100-150 x 10^9^/L )(7). The patients were treated by corticosteroids (11, comprising 5 with pulse), intravenous immunoglobulins (7), plasmapheresis (7), Rituximab (4) and cyclophosphamide (2). One patient died in ICU from pneumocystosis ; 5 are currently in complete remission and 5 in partial remission with a mean follow-up duration of 4,7 years.

## Conclusion

Generalized edema, digestive involvement (including abdominal pain), thrombocytopenia (with TMA), hyponatremia and hypoalbuminemia seem to be early warning signs of severe DMJ and should be identify to improve prognosis. Our study suggests that severe DMJ should early benefit from plasmapheresis ± Rituximab, whereas pulse corticosteroids might contribute to digestive perforation in patients presenting with digestive involvement.

## Disclosure of interest

None declared.

